# Association between the systemic immune-inflammation index and kidney stone: A cross-sectional study of NHANES 2007-2018

**DOI:** 10.3389/fimmu.2023.1116224

**Published:** 2023-02-21

**Authors:** Xingpeng Di, Shaozhuang Liu, Liyuan Xiang, Xi Jin

**Affiliations:** ^1^ Department of Urology, Institute of Urology (Laboratory of Reconstructive Urology), West China Hospital, Sichuan University, Chengdu, Sichuan, China; ^2^ Department of Urology, Shengjing Hospital of China Medical University, Shenyang, Liaoning, China; ^3^ Department of Clinical Research Management, West China Hospital, Sichuan University, Chengdu, Sichuan, China

**Keywords:** systemic immune-inflammatory index, kidney stone, neutrophil, lymphocyte, platelet, National Health and Nutrition Examination Survey

## Abstract

**Background:**

The incidence rate of kidney stones increased over the past decades globally, which brought medical expenditure and social burden. The systemic immune-inflammatory index (SII) was initially identified as a prognosis of multiple diseases. We performed an updated analysis on the impact of SII on kidney stones.

**Methods:**

This compensatory cross-sectional study enrolled participants from the National Health and Nutrition Examination Survey 2007-2018. Univariate and multivariate logistic regression analyses were performed to investigate the association between SII and kidney stones.

**Results:**

Of the 22220 participants, the mean (SD) age was 49.45 ± 17.36 years old, with a 9.87% incidence rate of kidney stones. A fully adjusted model showed that SII higher than 330 x 10^9^/L was parallel associated with kidney stones (Odds ratio [OR] = 1.282, 95% Confidence interval [CI] = 1.023 to 1.608, *P* = 0.034) in adults aged 20-50. However, no difference was found in the elderly subgroup. Multiple imputation analyses confirmed the robustness of our results.

**Conclusions:**

Our findings suggested SII was positively associated with a high risk of kidney stones in US adults aged less than 50. The outcome compensated for previous studies that still needed more large-scale prospective cohorts for validation.

## Introduction

In the past few decades, the prevalence of kidney stones increased worldwide. For instance, the incidence rate of kidney stones increased from 3.2% in the 1980s to 9.6% currently in the United States (US) (1, 2). Specifically, the incidence of symptomatic kidney stones is higher in the male population (3). For age stratification, the peak incidence rate in males is 40 to 60 years old in males, and 50 years old in females (4, 5). For the increasing trend of kidney stones, the risk factors include obesity, diabetes mellitus (DM), high intake of salt, animal protein, and added sugar (6–10). The health care of kidney stones has aroused great attention for the high medical expenditure and social burden (11).

Recently, kidney stone was reported to be associated with various inflammatory responses. For instance, C-reactive protein (CRP) concentration and the erythrocyte sedimentation rate (ESR) were identified as biomarkers for inflammatory diseases (12). In the systematic inflammatory system of the human body, immune cells are critical in multiple diseases. Several studies suggested that the neutrophil-to-lymphocyte ratio (NLR) was a crucial marker for kidney stone (13, 14). Moreover, studies demonstrated that platelet also engages in inflammatory response (15). The platelet-to-lymphocyte ratio was identified as an ideal predictor for systematic inflammatory response syndrome in patients undergoing percutaneous nephrolithotomy (PNL). Furthermore, researchers found that integrated peripheral lymphocyte, neutrophil, and platelet counts might be better to predict the inflammatory state, which also served as indicators for many diseases. The systemic immune-inflammatory index (SII) was initially identified as a prognosis of cancer, intracerebral hemorrhage, coronary stenosis, and others (16–18). However, the impact of SII on kidney stones is not fully elucidated, and little is known about its prognostic ability for kidney stones. For the relatively clarified association between NLR and PLR with kidney stone, we solely focused on SII in the current study. We hypothesized SII was a predictor for the risk of kidney stones. We performed the current study to investigate the association between SII and kidney stones.

## Methods

### Study population

**Figure 1:**
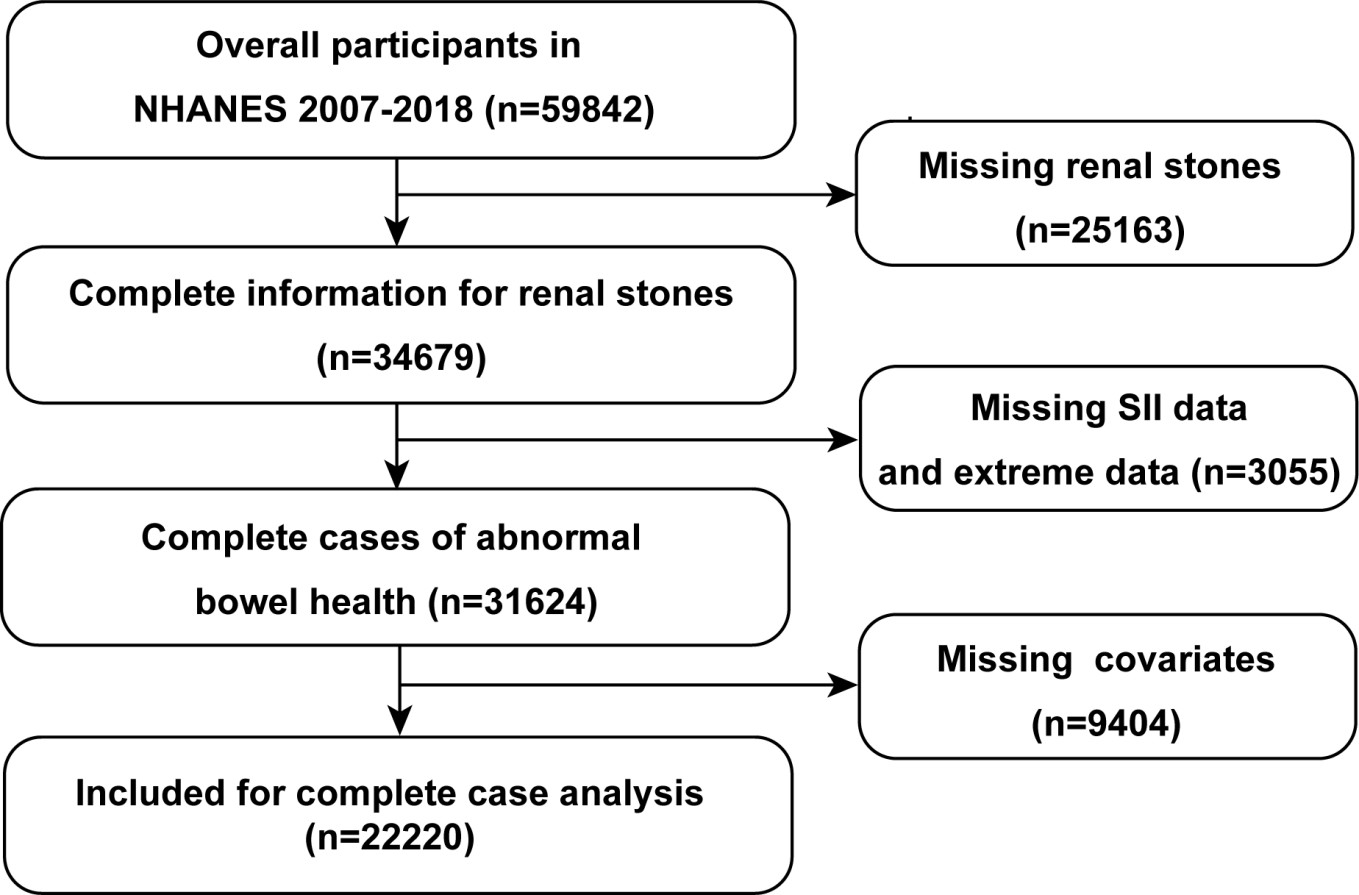
Overview of participants screening. NHANES, National Health and Nutrition Examination Survey; SII, systemic immune-inflammation index.

The current study was performed using a cross-sectional design of the NHANES dataset (19). Data from the NHANES database is collected every two years. All the protocols were approved by the National Center for Health Statistics institutional review board, and informed consent was required from participants. We enrolled 59842 participants from the year 2007 to 2018. The exclusion criteria included: (a) missing kidney stone data; (b) incomplete and extreme SII data (neutrophil, lymphocyte, and platelet count); (c) missing data of covariates. Finally, 2220 participants were included for complete case analysis (**Figure 1**).

### Assessment of kidney stone

Kidney stone history was defined by “Have you ever had kidney stones?” (2). The participants who reported an answer to the question indicated a history of diagnosed kidney stones.

### Assessment of systemic immune-inflammation index

The hypothesis of SII was first used by Hu et al. (20), to evaluate the prognostic value of multiple diseases. The SII was composed of peripheral neutrophil (N), lymphocyte (L), and platelet (P), which was defined as P x N/L (10^9^/L). We set the cutoff value at 330 x 10^9^/L for all subsequent analyses based on previous studies of the NHANES (21).

### Covariates definition

Participants self-reported age, gender, race/ethnicity, education level, family income-to-poverty ratio, smoking history, and alcohol drinking history (1, 22, 23). Race/ethnicity was divided into non-Hispanic Black, non-Hispanic White, Hispanic/Mexican, and other races. The family income-to-poverty ratio was classified into low (<1.3), median (1.3-3.5), and high (>3.5). Alcohol drinking history was defined as None (< 1 time per week), slight (1-3 times per week), and severe (to 4 times per week). The body mass index (BMI) was classified as lower than 20 kg/m^2^, 20-25 kg/m^2^, 25-30 kg/m^2^, and over 30 kg/m^2^. Smoking history, DM, and coronary heart disease were recorded by “Yes/No”. DM was diagnosed based on previous studies (24).

### Statistical analyses

The comparison in the SII subgroups was performed using survey-weighted logistic regression for continuous variables (mean ± standard deviation [SD]) and survey-weighted Chi-square test for categorical variables (counting number, [n]). Multivariate logistic regression analysis was conducted to investigate the relationship between SII and kidney stones. The crude model was adjusted for no covariates. Model 1 was a minimally-adjusted model adjusted for age, gender, race/ethnicity, family income-to-poverty ratio, and education level. Model 3 was adjusted for BMI, smoking history, alcohol drinking history, DM, and coronary heart disease. Age-stratified subgroup analysis was performed to clarify the impact of SII. Further stratified logistic regression analysis was conducted to identify variables that modify the association in participants aged 20-50 (25). Sensitivity analyses using the multiple imputation (MI) methods were performed. MI was an approach to compensate for the missing data based on five replications and a chained equation method in the *R* MI procedure to account for missing data on education level, family income-to-poverty ratio, BMI, smoking history, alcohol drinking history, DM, and coronary heart disease (26).

All analyses were performed utilizing *R* software version 4.1 (http://www.R-project.org; The R Foundation) and EmpowerStats (http://www.empowerstats.com, X&Y Solutions, Inc.). *P* < 0.05 (two-sided) was set for a significant difference.

## Results

There were 59842 participants from 2007-2018 included in this study. 25163 participants were excluded for missing kidney stone data, and 3054 participants were excluded for missing SII data. After removing 9405 participants missing covariates and extreme data for SII, 2220 participants were finally enrolled. Of the 22220 participants, there were 11755 males and 10465 females (**Table 1**). The prevalence rate of kidney stones was 9.87%. Compare with SII lower than 330 x 10^9^/L, participants with SII higher than 330 x 10^9^/L were more non-Hispanic White, smokers, DM, and kidney stones (*P* < 0.05).

**Table 1:** Basic characteristics of the study population in NHANES 2007-2018 (n = 22220).

Characteristics	Overall	SII (10^9^/L)		P value
		< 330	≥ 330	
**Number**	22220	5722	16498	
**Age**	49.45 ± 17.36	49.25 ± 17.24	49.52 ± 17.40	<0.001
**Family income-to-poverty ratio**	2.59 ± 1.64	2.58 ± 1.63	2.59 ± 1.64	0.363
**BMI (kg/m^2^)**	29.34 ± 7.04	28.47 ± 6.35	29.64 ± 7.25	<0.001
**Gender**				<0.001
Male	11755 (52.90%)	2414 (42.19%)	8051 (48.80%)	
Female	10465 (47.10%)	3308 (57.81%)	8447 (51.20%)	
**Race/Ethnicity**				<0.001
Non-Hispanic Black	4510 (20.30%)	1833 (32.03%)	2677 (16.23%)	
Non-Hispanic White	10171 (45.77%)	2000 (34.95%)	8171 (49.53%)	
Hispanic/Mexican	5276 (23.74%)	1234 (21.57%)	4042 (24.50%)	
Other Races	2263 (10.18%)	655 (11.45%)	1608 (9.75%)	
**Education level**				0.408
≤ High school	4752 (21.39%)	1239 (21.65%)	3513 (21.29%)	
> High school	17468 (78.61%)	4483 (78.35%)	12985 (78.71%)	
**Smoking history**				0.010
Non-smoker	11093 (49.92%)	3004 (52.50%)	8089 (49.03%)	
Smoker	11127 (50.08%)	2718 (47.50%)	8409 (50.97%)	
**Alcohol drinking history (drinks/week)**				0.156
< 1	14262 (64.19%)	3633 (63.49%)	10629 (64.43%)	
1-3	5645 (25.41%)	1500 (26.21%)	4145 (25.12%)	
≥ 4	2313 (10.41%)	589 (10.29%)	1724 (10.45%)	
**Diabetes mellitus**				<0.001
No	18067 (81.31%)	4709 (82.30%)	13358 (80.97%)	
Yes	4153 (18.69%)	1013 (17.70%)	3140 (19.03%)	
**Coronary heart disease**				0.090
No	21278 (95.76%)	5472 (95.63%)	15806 (95.81%)	
Yes	942 (4.24%)	250 (4.37%)	692 (4.19%)	
**Kidney stone**				0.010
No	20026 (90.13%)	5245 (91.66%)	14781 (89.59%)	
Yes	2194 (9.87%)	477 (8.34%)	1717 (10.41%)	

Data were n (%) or mean ± SD; BMI, body mass index; SII, systemic immune-inflammatory index.

Subsequently, logistic regression analysis found no significant difference between SII subgroups and kidney stones after adjusting for covariates in the whole population (**Table 2**). After the age was stratified by 50 years old, the univariate and multivariate analyses demonstrated high SII over 330 x 10^9^/L was associated with a higher risk of kidney stones in the crude model (Odds ratio [OR] = 1.485, 95% Confidence interval [CI] = 1.195 to 1.845, *P* < 0.001) and model 1 (OR = 1.387, 95% CI = 1.108 to 1.736, *P* = 0.005). After adjusting for all confounding factors, high SII was still positively associated with a kidney stone in the population aged 20-50 years old. (OR = 1.282, 95% CI = 1.023 to 1.608, *P* = 0.034) The difference was not found in participants aged 50 years old and above. Stratified logistic regression analysis in the 20-50 years old group suggested no potential modifiers in the relationship between SII and kidney stone in 20-50 population (**Supplementary Table S1**).

**Table 2:** Univariate and multivariate analyses by the activity-stratified logistic regression model, weighted.

Age stratification	SII (10^9^/L)		*P* value
	< 330 (OR, 95% CI)	≥ 330 (OR, 95% CI)	
Overall			
Crude model^a^	1.0 (Reference)	1.201 (1.043,1.383)	0.013
Model 1^b^	1.0 (Reference)	1.150 (0.995,1.328)	0.061
Model 2^c^	1.0 (Reference)	1.075 (0.928,1.246)	0.337
20-50			
Crude model	1.0 (Reference)	1.485 (1.195,1.845)	<0.001
Model 1	1.0 (Reference)	1.387 (1.108,1.736)	0.005
Model 2	1.0 (Reference)	1.282 (1.023,1.608)	0.034
≥ 50			
Crude model	1.0 (Reference)	1.036 (0.866,1.240)	0.701
Model 1	1.0 (Reference)	0.987 (0.820,1.188)	0.889
Model 2	1.0 (Reference)	0.938 (0.776,1.133)	0.506

a Crude model: adjusted for none.

b Model1: adjusted for age (only for overall), gender, race, education level, and family income-to-poverty ratio.

c Model2: adjusted for age, gender (only for overall), race, education level, family income-to-poverty ratio, BMI, smoking history, alcohol drinking history, DM, and coronary heart disease. P < 0.05 presents significant difference. BMI, body mass index; CI, confidence interval; DM, diabetes mellitus; OR, odds ratio; SII, systemic immune-inflammatory index.

For further validation of the outcomes, multiple imputations were performed. The baseline characters distribution was depicted in **Supplementary Table S2**. Sensitivity analysis demonstrated a similar result in the fully-adjusted model (OR = 1.251, 95% CI = 1.015 to 1.541, *P* = 0.036) (**Table 3**).

**Table 3:** The comparison between complete data analysis and multiple imputation analysis for detection of sensitivity.

Age	SII < 330 x 10^9^/L (OR, 95% CI)	SII ≥ 330 x 10^9^/L (OR, 95% CI), *P*	
		Complete case	Multiple imputation
20-50			
Model 2^a^	1.0 (Reference)	1.282 (1.023,1.608), 0.034	1.251 (1.015,1.541),0.036
≥ 50			
Model 2^a^	1.0 (Reference)	0.938 (0.776,1.133), 0.506	0.924 (0.785,1.088), 0.345

a Model 2: Adjusted for gender, race, education level, family income-to-poverty ratio, BMI, smoking history, alcohol drinking history, DM, and coronary heart disease. P < 0.05 presents significant difference. BMI, body mass index; DM, diabetes mellitus; OR, odds ratio; SII, systemic immune-inflammatory index.

## Discussion

The immune system has long been identified as a prognostic factor of multiple diseases. In this cross-sectional study, we identified higher SII level was independently associated with kidney stones in people lower than aged 50 years old. However, we did not find such an association in the elderly population. The relationship between SII and kidney stones was confirmed by multiple imputation sensitivity analyses. Importantly, the measurement of SII was based on a standard laboratory approach of peripheral neutrophils, lymphocytes, and platelet in clinical practice. Herein, SII can be identified as a biomarker for kidney stones in younger adults.

Several studies suggested the relationship between inflammatory biomarkers and diseases consistent with previous studies. A study investigated the prognostic value of SII on kidney stone former and passage from NHANES 2007-2014. The results demonstrated SII>444.37 indicated a higher incidence of kidney stones (27). However, our findings from NHANES 2007-2018 with updated data demonstrated that SII was the only indicator for kidney stones in the 20-50 years old group. Although there was a significant difference in all age groups, the difference disappeared after being adjusted for confounding factors.

Immune inflammation response was identified as engaging in multiple disease processes. A retrospective study compared the diagnostic values of leukocytes, neutrophils, neutrophil-to-lymphocyte (NLR), and platelet-to-lymphocyte (PLR) in distinguishing appendicitis and ureteral stones (28). The study identified NLR as a predictor in distinguishing appendicitis and ureteral stones. In addition, NLR and PLR were inversely associated with the spontaneous ureteral stone passage. However, a recent study demonstrated that inflammatory biomarkers such as NLR and PLR were not related to spontaneous ureteral stone passage (29). SII was recognized as a valuable and convenient inflammatory biomarker to predict the risk of low bone mineral density or osteoporosis among postmenopausal women aged ≥ 50 years old (25). Moreover, SII has also been reported concerning all-cause mortality in arteriosclerotic cardiovascular disease (30).

Recently, researchers suggested that multiple inflammatory processes engaged in kidney stone formation. Idiopathic calcium oxalate stones often attach to Randall’s plaque that was associated with the activation of M1 macrophages (31). While M2 macrophage-related genes are associated with the inhibition of stone formation (32). The renal crystal deposition is also related to reactive oxygen species (ROS) production, and inflammasome activation (33). The exosomes released by macrophage promoted the IL-8 production, facilitated neutrophil migration, and enhanced the crystal inflammatory response (34). Meanwhile, the exosomes reduced T-cell activation as well. For lymphocytes, a lower peripheral lymphocyte count indicated a higher SII level. Lymphocytes are critical components of leukocytes that mediate both innate and adaptive immune responses. A blood lymphocyte analysis in 36 kidney stones patients found three patients had lymphocyte depletion (35). In addition, platelets are increasingly identified as crucial modulators of inflammation response. Activated platelets trigger an intrinsic coagulation cascade that contributes to multiple diseases. Platelets can also accelerate inflammatory state (36). The platelet interacts with monocytes, neutrophils, and lymphocytes, and regulates innate and adaptive responses.

Interestingly, our findings only demonstrated the association between SII and kidney stones in people aged lower than 50. Indeed, obesity, older age, metabolic syndrome, and DM are all risk factors for diseases in the older population (37). Since there are more risk factors in older people than in younger ones, the impact of SII on kidney stones might be compensated by other confounding factors. In addition, for men aged lower than 60, a higher intake of calcium had a multivariate relative risk of 0.69 (38). However, no such trend was found in older people. Unfortunately, the relationship between the inflammatory markers and kidney stones was not able to be identified in the younger age group since the data for participants under 20 years old was unincluded. In addition, SII was reported to be an indicator of cardiovascular disease (39). Therefore, after the model was adjusted for DM and cardiovascular diseases in elderly people, the effect of SII was eliminated.

In the current study, we performed analyses to explore the association between SII and kidney stones based on the cross-sectional prospective NHANES dataset. Furthermore, adjustment for covariates allowed us to identify confounding factors that affected kidney stones. Our findings were validated by multiple imputation sensitivity analyses. Compare with the previous study, we updated the dataset to 2018 with a larger sample size and subgroup analysis to illustrate the risk of higher SII on kidney stone formation.

There were also some limitations. First, the cross-sectional design of the NHANES indicated that causal links cannot be established. Second, the interview forum for data collection may lead to potential bias. Third, some asymptomatic kidney stones without physical examination were missed in the database. Fourth, the immune cell count was obtained from one blood test only. Serial testing may be more reliable than one blood test on account of the blood cell life span. Finally, there were still some unobserved confounding factors that might be missed.

## Conclusion

This cross-sectional study suggested SII was positively associated with kidney stones in US adults aged less than 50. The outcome compensated for previous studies that still needed more large-scale prospective cohorts for validation.

## Data availability statement

Publicly available datasets were analyzed in this study. This data can be found here: NHANES database (https://www.cdc.gov/nchs/nhanes/index.htm).

## Author contributions

Conceptualization and Methodology: XJ and X-PD. Data curation and Project administration: X-PD and L-YX. Investigation and formal analysis:L-YX and S-ZL. Manuscript Writing- Original draft:X-PD. Manuscript editing and manuscript review:XJ and S-ZL. All authors contributed to the article and approved the submitted version.
