# Supplementary material for: Association between the systemic immune-inflammation index and kidney stone: A cross-sectional study of NHANES 2007-2018
Source: Front Immunol. 2023 Feb 21;14:1116224. doi: 10.3389/fimmu.2023.1116224 (PMC9989007; doi:10.3389/fimmu.2023.1116224)
Supplement: Supplementary file 1 [file Table_1.docx]

**Supplementary Table S1. Stratified logistic regression analysis to identify variables that modify the correlation between SII and kidney stone in participants aged 20-50, weighted.**

|  | **SII (10^9^**/L) | |  |
| --- | --- | --- | --- |
| **Characteristics** | **< 330 (OR, 95% CI)** | **≥ 330 (OR, 95% CI)** | ***P* for interaction** |
| **Gender** |  |  | 0.758 |
| Male | 1.0 (Reference) | 1.15 (0.84, 1.58) |  |
| Female | 1.0 (Reference) | 1.24 (0.89, 1.74) |  |
| **Race/Ethnicity** |  |  | 0.339 |
| Non-Hispanic Black | 1.0 (Reference) | 1.28 (0.77, 2.13) |  |
| Non-Hispanic White | 1.0 (Reference) | 1.23 (0.93, 1.64) |  |
| Hispanic/Mexican | 1.0 (Reference) | 0.92 (0.60, 1.41) |  |
| Other Races | 1.0 (Reference) | 1.78 (0.91, 3.45) |  |
| **Family income-to-poverty ratio** |  |  | 0.426 |
| <1.3 | 1.0 (Reference) | 1.27 (0.89, 1.80) |  |
| ≥ 1.3, <3.5 | 1.0 (Reference) | 1.03 (0.74, 1.44) |  |
| ≥ 3.5 | 1.0 (Reference) | 1.37 (0.96, 1.97) |  |
| **Education level** |  |  | 0.729 |
| ≤ High school | 1.0 (Reference) | 1.33 (0.73, 2.41) |  |
| > High school | 1.0 (Reference) | 1.19 (0.93, 1.52) |  |
| **BMI (kg/m^2^)** |  |  | 0.143 |
| ≤20 | 1.0 (Reference) | 3.12 (0.89, 10.92) |  |
| >20, ≤25 | 1.0 (Reference) | 0.94 (0.59, 1.50) |  |
| >25, ≤30 | 1.0 (Reference) | 1.09 (0.76, 1.58) |  |
| >30 | 1.0 (Reference) | 1.42 (1.02, 1.98) |  |
| **Smoking history** |  |  | 0.518 |
| Non-smoker | 1.0 (Reference) | 1.29 (0.94, 1.78) |  |
| Smoker | 1.0 (Reference) | 1.12 (0.82, 1.53) |  |
| **Alcohol drinking history (drinks/week)** |  |  | 0.206 |
| < 1 | 1.0 (Reference) | 1.19 (0.90, 1.57) |  |
| 1-3 | 1.0 (Reference) | 1.51 (0.97, 2.35) |  |
| ≥ 4 | 1.0 (Reference) | 0.66 (0.28, 1.54) |  |
| **Diabetes mellitus** |  |  | 0.676 |
| No | 1.0 (Reference) | 1.19 (0.93, 1.52) |  |
| Yes | 1.0 (Reference) | 1.40 (0.68, 2.91 |  |
| **Coronary heart disease** |  |  | 0.209 |
| No | 1.0 (Reference) | 1.20 (0.95, 1.51) |  |
| Yes | 1.0 (Reference) | 5.22 (0.52, 51.96) |  |

Adjusted for gender, race, education level, family income-to-poverty ratio, BMI, smoking history, alcohol drinking history, DM, and coronary heart disease. *P* < 0.05 presents significant difference. All the models are not adjusted for the variable itself in each stratification. BMI, body mass index; CI, confidence interval; DM, diabetes mellitus; OR, odds ratio, SII, systemic immune-inflammatory index.

**Supplementary Table S2. Distributions of variables with complete cases comparing to results from pooling the data with imputed variables from multiple imputation.**

| **Characteristics** | **Complete case** | **Multiple imputation** |
| --- | --- | --- |
| **Number** | 22220 | 31624 |
| **Age** | 49.45 ± 17.36 | 49.85 ± 17.69 |
| **Family income-to-poverty ratio** | 2.59 ± 1.64 | 2.48 ± 1.74 |
| **BMI (kg/m^2^)** | 29.34 ± 7.04 | 29.26 ± 4.17 |
| **Gender** |  |  |
| Male | 11755 (52.90%) | 15291 (48.35%) |
| Female | 10465 (47.10%) | 16333 (51.65%) |
| **Race/Ethnicity** |  |  |
| Non-Hispanic Black | 4510 (20.30%) | 6576 (20.79%) |
| Non-Hispanic White | 10171 (45.77%) | 13009 (41.14%) |
| Hispanic/Mexican | 5276 (23.74%) | 8138 (25.73%) |
| Other Race | 2263 (10.18%) | 3901 (12.34%) |
| **Education level** |  |  |
| ≤ High school | 4752 (21.39%) | 7802 (24.67%) |
| > High school | 17468 (78.61%) | 23822 (75.33%) |
| **Smoking history** |  |  |
| Non-smoker | 11093 (49.92%) | 17659 (55.84%) |
| Smoker | 11127 (50.08%) | 13965 (44.16%) |
| **Alcohol drinking history (drinks/week)** |  |  |
| < 1 | 14262 (64.19%) | 21036 (66.52%) |
| 1-3 | 5645 (25.41%) | 7556 (23.89%) |
| ≥ 4 | 2313 (10.41%) | 3026 (9.57%) |
| **Diabetes mellitus** |  |  |
| No | 18067 (81.31%) | 25412 (80.36%) |
| Yes | 4153 (18.69%) | 6212 (19.64%) |
| **Coronary heart disease** |  |  |
| No | 21278 (95.76%) | 30319 (95.87%) |
| Yes | 942 (4.24%) | 1305 (4.13%) |
| **Kidney stone** |  |  |
| No | 20026 (90.13%) | 28628 (90.53%) |
| Yes | 2194 (9.87%) | 2996 (9.47%) |

Data were n (%) or mean ± SD; BMI, body mass index.
